# The association of social isolation and family structure on stroke-specific quality of life in a community-based study

**DOI:** 10.1007/s11136-026-04337-2

**Published:** 2026-07-23

**Authors:** Josh Martins-Caulfield, Chen Chen, Lynda D. Lisabeth, Guanghao Zhang, Madeline Kwicklis, Leanna M. Delhey, Lewis B. Morgenstern

**Affiliations:** 1 Icahn School of Medicine at Mount Sinai, New York, NY USA; 2 Department of Neurology, University of Michigan Medical School, Ann Arbor, MI USA; 3 Department of Epidemiology, University of Michigan School of Public Health, 1415 Washington Heights, Ann Arbor, MI 48109 USA

**Keywords:** Social isolation, Quality of life, Loneliness, Stroke, Neurology

## Abstract

**Purpose:**

Quality of Life (QOL) is a critical patient-centered outcome for individuals who have experienced stroke. This study examined the association of social isolation and family structure with Stroke-Specific QOL (SSQOL).

**Methods:**

Participants were stroke survivors enrolled in the Brain Attack Surveillance in Corpus Christi (BASIC) study in Nueces County, Texas who completed 90-day post-stroke follow-up interviews between October 2019 and June 2023. Interviewers assessed QOL using the SSQOL scale, social isolation using the Patient-Reported Outcomes Measurement Information System (PROMIS) scale. Family structure was assessed based on marital status, family size, and whether adult children were living within 10 miles of the patient. Linear models were developed to analyze the relationship between social isolation and SSQOL with successive adjustment for demographic, clinical, socioeconomic, and familial variables from interviews and medical records.

**Results:**

Among 706 stroke survivors (median age = 64, 36% Non-Hispanic White and 64% Mexican American), median PROMIS social isolation score was 44 (IQR: 34.8–51.6) and median SSQOL was 3.67 (IQR: 2.75–4.50). The average family size was 2.18 people and 50% of participants were married while 60% had a child living within 10 miles of them. A consistent negative association between social isolation and SSQOL scores was observed across all adjusted models (fully adjusted $\beta =\hspace{0.17em}-\hspace{0.17em}$0.77, 95% CI = (− 0.88, − 0.65), *p* < 0.01), while family structure was not associated with SSQOL.

**Conclusions:**

Social isolation was negatively associated with SSQOL. There was no association between family structure and SSQOL. This study highlights the significance of modifiable psychosocial factors, such as social isolation, as potential key elements in stroke recovery.

## Abstract (plain language summary)

Neurologic disorders, including stroke, are increasing in incidence around the world as the population ages. Exploring factors that influence stroke survivor quality of is thus increasingly important as more people experience stroke. However, few studies have directly evaluated how psychosocial factors influence Stroke-Specific Quality of Life. This study examines how different psychosocial factors, including social isolation and family structure, affect stroke survivors’ quality of life. Social isolation and family structure were assessed through 90 day post stroke outcome interviews. This study found that social isolation was significantly associated with lower quality of life among stroke survivors. Measures of family structure (marital status, family size, and number of adult children living nearby) were not associated with changes in post-stroke quality of life. Interventions designed to decrease social isolation have the potential to make a substantial difference in the quality of life of stroke survivors.

## Introduction

The number of stroke survivors around the world has doubled over the past 30 years to 101 million people [1]. With the escalating number of stroke survivors, it is crucial to study the factors that may influence post-stroke recovery. Quality of Life (QOL) is an important patient-centered measure which is increasingly being employed to evaluate patient outcomes after illness because it offers a comprehensive assessment of illness repercussions [2, 3]. However, measuring QOL in stroke survivors is difficult due to stroke-specific physical and social sequelae [4]. One validated method to measure QOL in stroke survivors is the Stroke Specific Quality of Life Scale (SSQOL; range = 1–5, higher scores better) [4–7].

Understanding predictors of SSQOL is essential in shaping interventions to help improve stroke survivors’ lives. Previous research has indicated that social isolation, lower educational attainment, greater levels of disability, female sex, and being single are associated with lower QOL among stroke survivors, but few studies have reported on SSQOL [8–10].

One potential predictor of lower QOL among stroke survivors is social isolation. The association of loneliness with various health issues is well-documented, and many stroke survivors need personal caregiving in order to effectively recover [11, 12]. Commonly, those caregivers are family members [13]. Studies have shown that including family members as part of the recovery process and providing them education and resources helps stroke survivors recover and reduces their depressive symptoms [13, 14]. Further, understanding the association of social isolation and family structure on SSQOL is vital to determine how stroke survivors can best recover.

Our study sought to understand whether greater perceived social isolation is associated with worse SSQOL outcomes using a community-based cohort of Mexican American (MA) and non-Hispanic White (NHW) stroke survivors. We also sought to understand the impact of family structure on SSQOL. While research has been conducted on the association between psychosocial factors and various QOL outcomes, this study is among the first to examine the impact of social isolation on SSQOL specifically and in a diverse community-based setting. This investigation is critical for targeting interventions to improve stroke recovery experiences.

## Methods

### Participants

The Brain Attack Surveillance in Corpus Christi (BASIC) project is an ongoing population-based stroke surveillance project in Nueces County, Texas that began January 1, 2000. Detailed methods were previously published [15]. The county is a bi-ethnic, non-immigrant community of approximately 35% non-Hispanic White and 65% Mexican Americans and features little out-migration, providing a stable population for long-term outcome research.

The BASIC Project identifies all strokes in the county among residents aged 45 and older, using a combination of active and passive surveillance methods. Active surveillance includes reviewing hospital admission logs for stroke screening terms and monitoring hospital units for possible in-hospital strokes. Passive surveillance involves reviewing stroke-related diagnostic codes in hospital and emergency department discharge records. Possible strokes undergo validation by a stroke fellowship-trained physician masked to ethnicity and age. Stroke survivors or their proxies are then recruited to participate in baseline interviews, most often done within the first few weeks after stroke. Interviews are available in both Spanish and English. Around ninety days post-stroke, surviving participants are invited to another interview assessing outcomes. In addition, clinical information from the stroke hospitalization and demographics for all eligible survivors are abstracted from medical records. A detailed description of BASIC project methods is available elsewhere [16]. This analysis focused on a subset of individuals from the BASIC Project who experienced either intracerebral hemorrhage (ICH) or ischemic stroke from October 1, 2019, to June 1, 2023, and survived for at least 90 days (n = 2046).

The STROBE Research Guidelines reporting guidelines were followed.

### Variables

The outcome variable of interest is the SSQOL Short Form score, which uses a scale of 1–5 with higher scores indicating better function (as the average of the 12 SSQOL items) [7, 17]. The main exposure is the Patient-Reported Outcomes Measurement Information System (PROMIS) Social Isolation 4a T-score, a four-item scale ranging from 34.8 to 74.2, using the T-score conversion table, with higher scores indicating more isolation and 50 being the population mean social isolation score [18]. Additionally, family structure was assessed through three variables: marital status, family size, and adult children living within 10 miles. The social isolation score was collected at 90 days and was only available for interviews completed by patients. We additionally collected data on covariates from baseline interviews and medical records including demographic variables (age, sex, and ethnicity), clinical variables (Tissue Plasminogen Activator (tPA), initial National Institutes of Health Stroke Scale (NIHSS), number of comorbidities, pre-stroke disability from the baseline interview, pre-stroke cognition, and pre-stroke depression). Pre-stroke disability was measured by the modified Rankin Score (mRS), and pre-stroke cognitive decline was measured by the Informant Questionnaire on Cognitive Decline in the Elderly (IQCODE). Other variables derived from baseline interview questions included socioeconomic status (education and family income) and familial variables (marital status, whether the patient has adult children who live within 10 miles, and family size). Family size—the number of adults living in the household —was treated as a continuous variable in subsequent analyses. Marital status was binarized as married/living with a partner versus unmarried.

### Statistical analysis

**Fig. 1:**
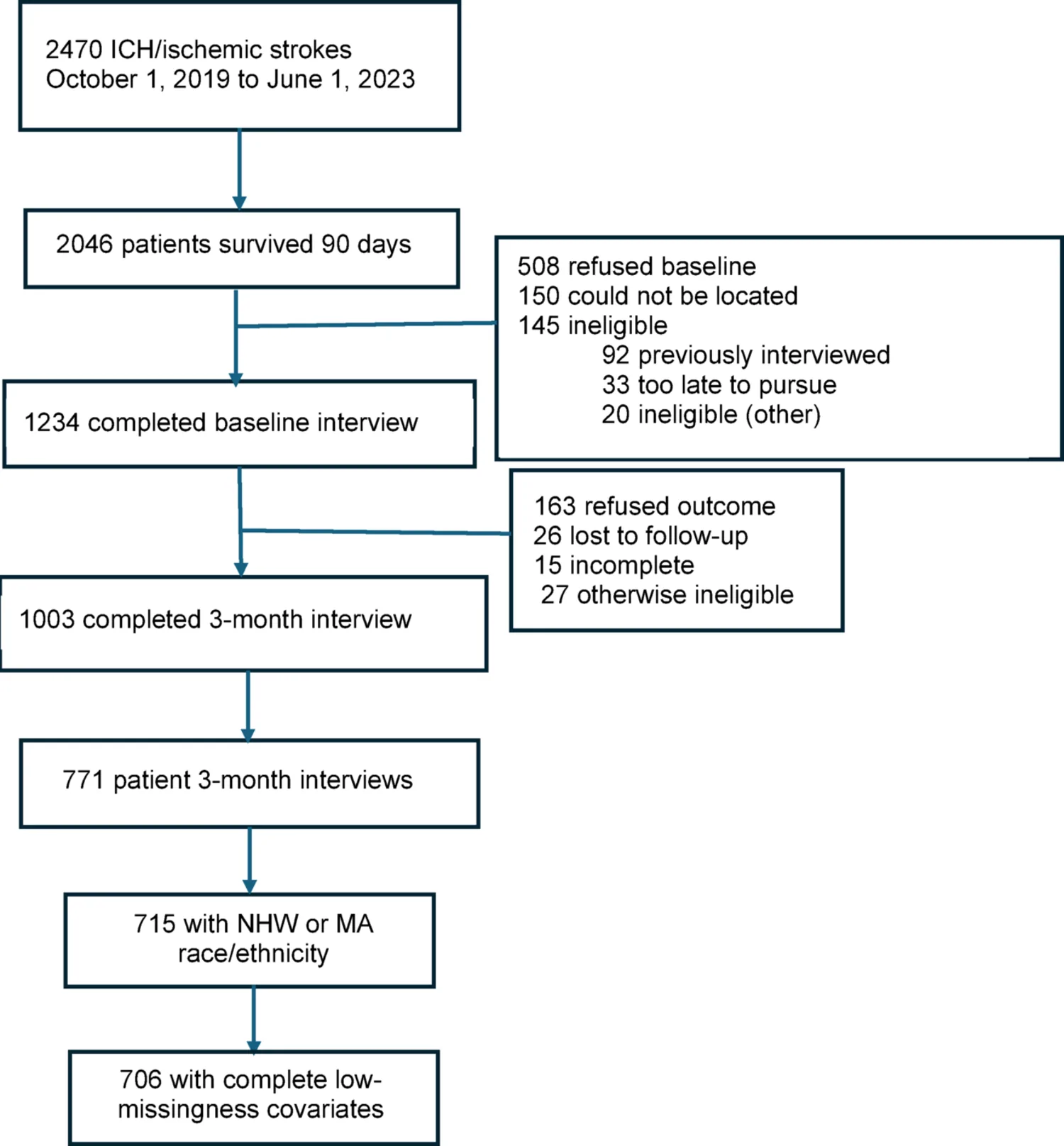
Data flow chart. This figure shows the flow of participants through the study

We analyzed the relationship between social isolation scores and SSQOL using weighted linear regression models. Covariates with higher rates of missingness, including IQCODE, depression, and income, were multiply imputed with chained equations using the R package mice version 3.16.0 and results were pooled across 20 imputed datasets using Rubin’s rules [19]. To correct for potential participation biases at different stages (baseline participation, outcome interview participation, and patient-completed outcome interview, see Fig. 1), we developed three sets of weights within each imputed dataset by inverting propensity scores from logistic regression models, incorporating demographics and medical record data at the tier corresponding to baseline attrition, and then adding predictors from the baseline interview to the models corresponding to outcome and non-proxy interview attrition. The final weights for the regression analysis were obtained by multiplying these three sets of weights together and trimmed at the 2nd and 99th percentiles.

Our analysis included four sequential models to assess the impact of different types of covariates on the primary association. In Model 1, we only included the social isolation score and demographic variables. In Model 2, we further added clinical variables. In Model 3, we further included socioeconomic variables. In the fully adjusted Model 4, we further included familial variables. The functional form of social isolation was assessed through residual diagnostics and deemed to be linear throughout its interquartile range. Isolation was included in linear form, and the expected difference between the 75th and 25th percentiles of isolation was reported for each model. The weighted linear regression models were fitted using R package *survey* version 4.4-2 [20].

A parallel set of sequential models was used to evaluate the impact of the covariates on the associations between family structure variables and SS-QOL. All three family structure variables were simultaneously incorporated in Model 1, with demographics. Clinical variables were added in Model 2, and socioeconomic variables in Model 3. Model 4 was exactly equivalent to the Model 4 described for social isolation. The functional form of family size was evaluated through residual diagnostics; family size was included as a linear term.

A post-hoc analysis assessed whether family structure and social isolation were associated with physical aspects of QOL to the same extent as mental aspects. SS-QOL was split into its psychosocial and physical domains [16]. Two separate linear regression models for each subdomain were used to estimate the effects of family structure and social isolation, adjusting for the other exposures in addition to full covariate adjustment.

### Human subjects

The University of Michigan Medical School and Christus Health Institutional Review Boards approved all study procedures, and informed consent was obtained from all participants or their proxies. The IRB registration number is IRB00000244 and last approval date was 8/21/2025. Author LM had full access to all the data in the study and takes responsibility for its integrity and the data analysis.

## Results

A total of 2470 stroke patients were identified from October 1, 2019 to June 1, 2023 and 2046 survived 90 days. Of those, 508 refused to participate in the baseline interview, 150 could not be located, and 145 were ineligible. This left 1234 patients and proxies who completed the baseline interview, of which 1003 completed the 90 day follow up interview. Data from proxies were removed from analysis leaving a study population of 771 stroke survivors. Participants with non-NHW or MA race/ethnicity or who were missing in any variables with overall high (>1%) missingness (medical record variables, education, marital status, pre-stroke disability, and demographics) were excluded in the main analysis, resulting in a final study sample of 706. Figure 1 shows the flow of participants through the study.

Participant characteristics are provided in Table 1. The mean social isolation score was 44.0 (SD = 9.4), which is below the population mean T-score of 50. The mean SSQOL score was 3.60 (SD = 0.97). The stroke survivors in this cohort ranged from 45 to 95 years old with an average age of 64. The cohort closely approximated the demographic composition of the surrounding community in regard to ethnicity (36% Non-Hispanic White (NHW) and 64% Mexican American (MA)), sex (47% female, 53% male), and educational attainment (47% had some post-secondary education; 28% were high school graduates; 25% did not graduate from high school) [21]. About half of participants were married, the average family size was 2.18, and 60% had a child living within 10 miles of them. At baseline, 19% received tPA treatment, the average number of comorbidities was 2.72, the average initial NIHSS score was 4.23, and the average Rankin score for pre-stroke disability was 1.56. At the baseline interview, about 62% reported no previous depression diagnosis, 18% were previously diagnosed or treated, and 20% were currently being treated for depression.

**Table 1:** The study population characteristics

Variables	The study population (N = 706) Mean (SD) [Range]/N(%)	% missing
Age	64.19 (10.59)	0%
*Sex*		0%
Male	373 (52.83%)	
Female	333 (47.17%)	
*Ethnicity*		0%
NHW	253 (35.84%)	
MA	453 (64.16%)	
*TPA*		0%
No	571 (80.88%)	
Yes	135 (19.12%)	
Initial NIHSS	4.23 (4.93)	0%
Number of comorbidities	2.72 (1.62)	0%
Baseline Rankin	1.56 (1.36)	0%
*Depression*		3.5%
Not diagnosed	421 (61.82%)	
Ever diagnosed /prescribed	123 (18.06%)	
Currently prescribed	137 (20.12%)	
IQCODE	3.19 (0.36)	17.0%
*Education*		0%
Less than high school	173 (24.50%)	
High school	199 (28.19%)	
More than high school	334 (47.31%)	
*Family income*		7.8%
< $10,000	120 (18.43%)	
$10,000 to $19,999	130 (19.97%)	
$20,000 to $29,999	99 (15.21%)	
$30,000 to $49,999	97 (14.90%)	
> $50,000	205 (31.49%)	
*Marital status*		0%
Not married	356 (50.42%)	
Married	350 (49.58%)	
Family size	2.18 (1.07)	0%
*Adult kid in 10 miles*		0%
No	279 (39.7%)	
Yes	425 (60.37%)	

This table provides the study population characteristics and amount of missing data. Abbreviations: NHW = Non-hispanic white; MA = Mexican American; TPA = Tissue plasminogen activator; NIHSS = NIH stroke scale; IQCODE = Informant questionnaire on cognitive decline in the elderly

Across all models shown in Table 2, social isolation was significantly negatively associated with SSQOL (*p* < 0.01 for all models, fully adjusted $\beta =\hspace{0.17em}-\hspace{0.17em}$0.75, 95% CI = (− 0.86, − 0.63)). The association between variables representing family structure and SSQOL is shown in Table 3. In the fully adjusted models, marital status (*β * = − 0.12, 95% CI = (− 0.25, 0.02), *p* = 0.08), family size (*β * = − 0.00, 95% CI = (− 0.06, 0.07), *p* = 0.93), and having a child within 10 miles (*β * = − 0.11, 95% CI = (− 0.24, 0.03), *p* = 0.12) were not significantly associated with SSQOL.

**Table 2:** Main analysis results—social isolation

Variables	Model 1: adjusted for demographic variables^1^		Model 2: Model 1 + adjusted for clinical variables^2^		Model 3: Model 2 + adjusted for socioeconomic variables^3^		Model 4: MODEL 3 + adjusted for familial variables^4^	
	Beta (95%CI)	*P* value	Beta (95%CI)	*P* value	Beta (95%CI)	*P* value	Beta (95%CI)	*P* value
Social isolation score (75th vs 25th percentile)	− 0.96 (− 1.08, − 0.84)	< 0.01	− 0.75 (− 0.87, − 0.63)	< 0.01	− 0.74 (− 0.86, − 0.63)	< 0.01	− 0.75 (− 0.86, − 0.63)	< 0.01
Age	0.00 (− 0.01, 0.00)	0.27	0.00 (0.00, 0.01)	0.31	0.00 (0.00, 0.01)	0.34	0.00 (− 0.00, 0.01)	0.31
*Sex*								
Male	(Reference)							
Female	− 0.08 (− 0.22, 0.06)	0.26	0.01 (− 0.11, 0.13)	0.84	0.03 (− 0.09, 0.15)	0.66	0.03 (− 0.09, 0.15)	0.64
*Ethnicity*								
NHW	(Reference)							
MA	− 0.30 (− 0.45, − 0.16)	< 0.01	− 0.14 (− 0.26, − 0.01)	0.03	− 0.06 (− 0.20, 0.08)	0.40	− 0.04 (− 0.18, 0.10)	0.61
*TPA*								
No	(Reference)							
Yes			0.27 (0.13, 0.42)	< 0.01	0.26 (0.11, 0.41)	< 0.01	0.27 (0.12, 0.42)	< 0.01
Initial NIHSS (log-transformed)			− 0.16 (− 0.23, − 0.08)	< 0.01	− 0.15 (− 0.22, − 0.08)	< 0.01	− 0.16 (− 0.23, − 0.09)	< 0.01
Number of comorbidities			− 0.09 (− 0.13, − 0.05)	< 0.01	− 0.08 (− 0.12, − 0.04)	< 0.01	− 0.08 (− 0.12, − 0.05)	< 0.01
Pre-stroke Rankin			− 0.19 (− 0.24, − 0.14)	< 0.01	− 0.18 (− 0.23, − 0.12)	< 0.01	− 0.18 (− 0.23, − 0.12)	< 0.01
*Depression*								
Not diagnosed	(Reference)							
Previously diagnosed or treated			− 0.02 (− 0.19, 0.15)	0.80	− 0.05 (− 0.22, 0.12)	0.57	− 0.07 (− 0.24, 0.09)	0.42
Currently treated			− 0.23 (− 0.42, − 0.05)	0.01	− 0.23 (− 0.41, − 0.05)	0.01	− 0.23 (− 0.41, − 0.05)	0.01
*IQCODE*								
3.01–3.43			− 0.02 (− 0.17, 0.12)	0.78	− 0.01 (− 0.15, 0.13)	0.91	0.00 (− 0.14, 0.14)	1.00
> 3.43			− 0.11 (− 0.31, 0.09)	0.29	− 0.07 (− 0.27, 0.13)	0.49	− 0.08 (− 0.27, 0.12)	0.44
*Education*								
Less than high school	(Reference)							
High school					0.06 (− 0.12, 0.24)	0.49	0.06 (− 0.12, 0.24)	0.53
More than high school					0.16 (− 0.01, 0.33)	0.07	0.15 (− 0.02, 0.32)	0.08
*Family income*								
< $10,000	(Reference)							
$10,000 to $19,999					0.00 (− 0.19, 0.18)	0.96	0.00 (− 0.18, 0.19)	0.96
$20,000 to $29,999					− 0.03 (− 0.23, 0.17)	0.76	0.00 (− 0.10, 0.20)	0.99
$30,000 to $49,999					0.09 (− 0.14, 0.31)	0.44	0.14 (− 0.08, 0.36)	0.22
> $50,000					0.15 (− 0.06, 0.36)	0.17	0.20 (− 0.02, 0.42)	0.07
*Marital status*								
Not married	(Reference)							
Married							− 0.12 (− 0.25, 0.02)	0.08
Family size							0.00 (− 0.06, 0.07)	0.93
*Adult child in 10 miles*								
No	(Reference)							
Yes							− 0.11 (− 0.24, 0.03)	0.12

This Table provides the main analysis results with four consecutive models showing the relationship between social isolation and SSQOL adjusted for various variables outlined by the footnotes. 1: Demographic variables include age, gender, and ethnicity

2: Clinical variables include baseline TPA, initial NIHSS, comorbidity score, pre-stroke Rankin, and depression

3: Socioeconomic variables include education and family income

4: family variables include marital status, family size, and whether has adult kids in 10 miles

Abbreviations: NHW = Non-hispanic white; MA = Mexican American; TPA = Tissue plasminogen activator; NIHSS = NIH stroke scale; IQCODE = Informant questionnaire on cognitive decline in the elderly

**Table 3:** Main analysis results—family structure

Variables	Model 1: adjusted for demographic variables^1^		Model 2: Model 1 + adjusted for clinical variables^2^		Model 3: Model 2 + adjusted for socioeconomic variables^3^	
	Beta (95%CI)	*P* value	Beta (95%CI)	*P* value	Beta (95%CI)	*P* value
*Marital status*						
Not married	(Reference)					
Married	0.20 (0.03, 0.38)	0.02	0.04 (− 0.10, 0.18)	0.60	− 0.03 (− 0.17, 0.12)	0.64
Family size	0.04 (− 0.05, 0.11)	0.34	0.02 (− 0.05, 0.09)	0.65	0.00 (− 0.06, 0.07)	0.91
*Adult child in 10 miles*						
No	(Reference)					
Yes	− 0.11 (− 0.32, 0.05)	0.15	− 0.16 (− 0.31, − 0.00)	0.05	− 0.14 (− 0.29, − 0.01)	0.06
Social isolation score (75th vs 25th percentile)	–	–	–	–	–	–
Age	0 (− 0.01, 0.01)	0.99	0.01 (0.0, 0.01)	0.06	0.01 (− 0.00, 0.01)	0.13
*Sex*						
Male	(Reference)					
Female	− 0.14 (− 0.31, 0.03)	0.10	0.01 (− 0.13, 0.15)	0.86	0.02 (− 0.12, 0.16)	0.74
*Ethnicity*						
NHW	(Reference)					
MA	− 0.34 (− 0.52, − 0.16)	< 0.01	− 0.11 (− 0.26, 0.03)	0.13	− 0.04 (− 0.20, 0.12)	0.61
*TPA*						
No	(Reference)					
Yes	–	–	0.31 (0.16, 0.47)	< 0.01	0.29 (0.14, 0.45)	< 0.01
Initial NIHSS (log-transformed)	–	–	− 0.20 (− 0.29, − 0.11)	< 0.01	− 0.21 (− 0.29, − 0.12)	< 0.01
Number of comorbidities	–	–	− 0.10 (− 0.14, − 0.06)	< 0.01	− 0.09 (− 0.13, − 0.05)	< 0.01
Pre-stroke Rankin	–	–	− 0.23 (− 0.29, − 0.18)	< 0.01	− 0.21 (− 0.27, − 0.15)	< 0.01
*Depression*						
Not diagnosed	(Reference)					
Previously diagnosed or treated	–	–	− 0.25 (− 0.43, − 0.07)	< 0.01	− 0.29 (− 0.46, − 0.10)	< 0.01
Currently treated	–	–	− 0.40 (− 0.59, − 0.22)	< 0.01	− 0.40 (− 0.58, − 0.22)	< 0.01
*IQCODE*						
3.01–3.43	–	–	0.02 (− 0.15, 0.18)	0.84	0.03 (− 0.13, 0.19)	0.70
> 3.43	–	–	− 0.12 (− 0.34, 0.11)	0.30	− 0.08 (− 0.29, 0.14)	0.50
*Education*						
Less than high school	(Reference)					
High school	–	–	–	–	− 0.01 (− 0.21, 0.19)	0.93
More than high school	–	–	–	–	0.07 (− 0.12, 0.25)	0.47
*Family income*						
< $10,000	(Reference)					
$10,000 to $19,999	–	–	–	–	0.03 (− 0.18, 0.24)	0.78
$20,000 to $29,999	–	–	–	–	0.07 (− 0.15, 0.28)	0.56
$30,000 to $49,999	–	–	–	–	0.11 (− 0.14, 0.36)	0.37
> $50,000	–	–	–	–	0.34 (0.11, 0.57)	< 0.01

This table provides the models for family structure’s association with SSQOL. The models are adjusted for the same variables as Table 2. 1: Demographic variables include age, gender, and ethnicity

2: Clinical variables include baseline TPA, initial NIHSS, comorbidity score, pre-stroke Rankin, and depression

3: Socioeconomic variables include education and family income

Abbreviations: NHW = Non-hispanic white; MA = Mexican American; TPA = Tissue plasminogen activator; NIHSS = NIH stroke scale; IQCODE = Informant questionnaire on cognitive decline in the elderly

Table 4 provides how social isolation and the family structure variables are associated with the subdomains of physical (mean = 4.05, SD = 0.86) and psychosocial (mean = 3.17, SD = 1.27) SSQOL. Marital status was not significantly associated with either component of SSQOL but trended negatively with the psychosocial component (*β * = − 0.16, 95% CI = (− 0.33, 0.01), *p* = 0.06), after adjustment for social isolation and other covariates. Family size (*p* = 0.86 for physical, 0.75 for psychosocial) and adult child within 10 miles (*p* = 0.29 for physical, 0.13 for psychosocial) were not independently associated with either subdomain. Social isolation was negatively associated with both subdomains, more strongly with the psychosocial component of SSQOL (psychosocial *β * = − 1.29, 95% CI = (− 1.48, − 1.10), *p*-value < 0.01; than the physical component *β *= − 0.47, 95% CI = (− 0.65, − 0.30), *p*-value < 0.01).

**Table 4:** Fully adjusted SSQOL subdomain results

Variables	Physical subdomain		Psychosocial subdomain	
	Beta (95% CI)	*p*-value	Beta (95% CI)	*p*-value
Marital status: married	− 0.07 (− 0.23, 0.08)	0.35	− 0.16 (− 0.33, 0.01)	0.06
Family size	0.01 (− 0.06, 0.07)	0.86	0.01 (− 0.07, 0.1)	0.75
Adult kid in 10 miles	− 0.08 (− 0.21, 0.06)	0.29	− 0.13 (− 0.3, 0.04)	0.13
Social isolation score (75th vs 25th percentile)	− 0.47 (− 0.65, − 0.3)	< 0.01	− 1.29 (− 1.48, − 1.1)	< 0.01

Adjusting for demographics, tPA, initial NIHSS, number of comorbidities, pre-stroke disability and cognition, depression, education, and income

This table shows the associations of family structure variables and social isolation score with each subdomain (physical or psychosocial) of SSQOL

## Discussion

This study demonstrates a consistent association between social isolation and lower SSQOL at 90 days among stroke survivors after adjusting for demographic, clinical, socioeconomic, and familial factors. This analysis adds to findings of similar studies, which collectively illustrate that adverse post-stroke outcomes are frequently correlated with social isolation, as well as other related conditions such as loneliness, and dissatisfaction with the level of social support [8–10, 22, 23]. Notably, this research is among the first to identify this association within a diverse, stable community. Identifying this association could assist providers and community leaders to develop community-specific resources to mitigate the effects of social isolation on stroke recovery and potentially other health outcomes. The findings in this paper contribute to the growing consensus of the scientific community positioning social isolation as a substantial public health concern.

Additionally, this study is one of the first to examine the association of social isolation with SSQOL, rather than other general QOL measures. The SSQOL is validated to provide a more precise assessment of recovery for stroke patients than other measures of QOL [4, 5, 7, 17]. In our fully adjusted model, the SSQOL score decreased by 0.75 points comparing higher versus lower social isolation score based on the IQR. This is a large and clinically meaningful decline given the 1–5 range of the SSQOL scale (s.d. 0.97). These results had interesting parallels compared to studies that measured QOL in stroke survivors with other measures. For example, two studies that used the Quality of Life Index (QLI) found moderate effect sizes (*β =* + 0.27 and + 0.3) for the impact of social support on QOL [9, 10]. While we cannot make direct comparisons with this model since the scales are not standardized, that result is congruent with our 0.75 point reduction in SSQOL with social isolation. However, the FRAILTY study found no association between perceived social support with health-related quality of life (HRQoL) in younger people (aged 18–65) [24]. Both this study and the FRAILTY study cannot prove causality, however the seemingly different results could point to varying importance of social isolation on SSQOL depending on age. Another consideration is that perhaps the HRQoL measure the FRAILTY study used may not have captured the role that social isolation plays in the recovery of stroke survivors specifically, compared to the use of SSQOL in this study.

While we found social isolation to be associated with worse SSQOL across all models, the greatest impact to the strength of the association came after adjusting for clinical variables. The effect size decreased in magnitude by nearly 22% from Model 1 to Model 2 and remained at around − 0.75 in Models 3 and 4. This could suggest that clinical variables such as stroke severity, disability, and comorbidities partially confound the relationship between social isolation and SSQOL. It is reasonable that more severe strokes could lead to greater social isolation among some patients, influencing the association with SSQOL. After controlling for these variables, however, the independent effect of social isolation was smaller but still substantial.

Table 4 shows the association of social isolation with SSQOL broken down into its psychosocial and physical subdomains. Social isolation was found to be significantly associated with worse scores in both SSQOL subdomains, however it had a larger impact on the psychosocial score compared to physical. This means that while social isolation significantly affects a stroke survivor’s physical and emotional recovery, its effect is almost three times larger on emotional well-being.

There are various ways social isolation can interact with both subdomains. For instance, it is reasonable that more isolated individuals have worse social support networks, negatively affecting their psychosocial SSQOL. Additionally, it can also be reasoned that isolation can affect an individual’s physical subdomain of SSQOL if they lack help in physical tasks in their lives. However, it is important to note that SSQOL is a multidimensional construct made up of many individual components, making analysis of individual components less informative. Nonetheless, the observed associations have implications for addressing gaps in stroke recovery. It is well-established that loneliness and isolation is a public health problem; addressing this in stroke survivors is crucial to helping them live their best lives after experiencing a stroke [11, 22, 23, 25]. Establishing support groups, social programs, and helping create connections for survivors is an important direction for stakeholders to explore in stroke recovery.

In contrast to social isolation, family structure as measured by marital status, family size, and children living within 10 miles was not independently associated with SSQOL after adjustment for demographic, clinical, and socioeconomic factors. In fact, while marital status initially appeared to be associated with higher SSQOL in our simplest model, adjustment for clinical and socioeconomic variables actually reversed this association. This could suggest that any potential advantages that being married brings to SSQOL is explained by better health, functional status, and/or socioeconomic resources. Interestingly, marital status was not associated with the physical domain of SSQOL, but trended towards a negative association (although still insignificantly) with the psychosocial domain. These findings suggest that the subjective perception of social isolation among stroke survivors is more important to the psychosocial aspects of QOL.

Strengths of this study include identification of psychosocial variables contributing to stroke recovery in a bi-ethnic, non-immigrant, and geographically stable community enabling a more accurate assessment of outcomes in this community. Additionally, the BASIC project has been operating in this community for over 25 years and has developed a validated framework for stroke surveillance and data collection. By using a patient-reported outcome, this study applies methodological rigor to recognize the lived experiences of stroke survivors.

Limitations include this study’s observational design which limits inference of causality between social isolation and SSQOL. Additionally, self-reported data introduces risk of recall and reporting bias. Generalizability may be limited to non-immigrant MAs rather than a broader population of Hispanic/Latinx. The study also took place during the COVID-19 pandemic, which may explain the relatively low participation rate and could affect findings. While the inverse probability weighting applied in this analysis can moderate bias due to sample selection, it cannot account for attrition driven by unobserved variables or the outcomes themselves. For example, the association between isolation and QOL might be stronger or weaker for the sickest patients who improve least, and who may be more likely to exit the sample through death and non-participation in the interviews, thus biasing the estimate in the final sample. Social isolation and the SSQOL were measured at the same time, which could result in reverse causality. Finally, the analysis used outcome data from a single time point of 90 days post stroke, limiting insight into long-term trends.

## Conclusions

Social isolation is consistently associated with lower SSQOL in stroke survivors, after adjusting for demographic, clinical, socioeconomic, and familial variables, which has implications for interventions to improve stroke recovery.

## Data Availability

Deidentified data for this paper may be requested from the corresponding author by a qualified investigator.
